# Coverage of skin cancer and recreational tanning in North American magazines before and after the landmark 2006 International Agency for Research on Cancer report

**DOI:** 10.1186/s12889-015-1511-1

**Published:** 2015-02-21

**Authors:** Jennifer E McWhirter, Laurie Hoffman-Goetz

**Affiliations:** School of Public Health and Health Systems, University of Waterloo, 200 University Avenue W, Ontario, Canada, N2L 3G1

**Keywords:** Skin cancer, Melanoma, Indoor tanning, Mass media, Health communication, UV protection, Sunscreen, Early detection, Prevention, Content analysis

## Abstract

**Background:**

Skin cancer is an increasingly important global public health problem. Mass media is a key source of skin cancer information. We examined how media coverage of skin cancer has changed over time as a consequence of the release of a key public health report from the International Agency for Research on Cancer (IARC) in 2006, which linked ultraviolet (UV) radiation from indoor tanning and skin cancer.

**Methods:**

A directed content analysis of skin cancer and tanning coverage in 29 popular North American magazines (2001–2012) examined reporting of skin cancer risk factors, UV behaviors, and early detection in article text (*n* = 761) and images (*n* = 1267). Chi-square and correlational analyses were used determine whether coverage changed in relation to the 2006 IARC report.

**Results:**

The total volume of articles about skin cancer and tanning increased modestly after the IARC report (*χ*^2^ = 4.57, *df* = 1, *p* < .05); however, key IARC report messages (e.g., avoid indoor tanning, UV as a risk factor) were no more likely to be reported after compared to before the report. There were virtually no changes in the percentage of coverage for both risk factors and early detection information over time. There were some changes in the percentage of coverage about UV behaviors after the IARC report, but these variables were not directly related to the report. Magazines were more likely to encourage sunscreen use (*χ*^2^ = 11.55, *df* = 1, *p* < .01) and less likely to promote the tanned look as attractive (*χ*^2^ = 9.72, *df* = 1, *p* < .01) after the IARC report. It also became less common for magazines to promote sun avoidance (*χ*^2^ = 6.82, *df* = 1, *p* < .01) and use of sunless tanners (*χ*^2^ = 7.46, *df* = 1, *p* < .01) after the report.

**Conclusions:**

Despite a modest increase in volume of coverage post-IARC report, key messages from the report were not taken up by the media. While there have been some improvements in magazine reporting, there is a need for more effective dissemination of public health messages about skin cancer and tanning.

## Background

Skin cancer is a significant public health problem globally [1], with incidence increasing in North America [2,3]. Skin cancer can often be prevented through modifiable behaviors [4] and, when detected early, survival for melanoma and non-melanoma is as high as 90% [5].

Ultraviolet (UV) radiation exposure, either solar or artificial, is *the* major risk factor for skin cancer [4,6]. UV exposure can be minimized through changes in behavior, such as avoiding excessive exposure to the sun, not using indoor tanning beds, wearing protective clothing and a wide-brimmed hat, seeking shade, and wearing sunscreen [4]. Despite this, indoor tanning is popular, especially among young Caucasian women in the United States, Europe, and Australia [7,8].

The mass media is an important source of health information for the public [9], influencing people’s attitudes and beliefs about skin cancer and tanning [10]. Moreover, media advocacy has a significant role in shaping the policy agenda around indoor tanning [11]. A handful of studies have examined temporal changes in media coverage of skin cancer and tanning, but the direction of changes over time has not been consistent. For example, between the 1997 and 2006, there was an increase in mentions of the consequences of UV exposure, but also an increase in coverage of the presumed “health” benefits of tanning in American women’s magazines [12]. In contrast, there was no change in media attention to skin cancer in American newspapers between 1986 and 2003 [13]. Still another study found a slight upward trend in news coverage between 1980 and 2004, but with considerable fluctuation from year to year; the largest increases in coverage occurred in the early and mid-1980s when several national skin cancer programs were established [14]. More recently, researchers found there was no change in the frequency of articles focusing on UV protection issues in Australian news media between 2001 and 2012 and an increase in articles focusing on sunbed issues between 2001 and 2005 [15]. While these studies make important contributions to knowledge about mass media reporting on skin cancer and tanning, research gaps remain: there has been no research on North American magazine coverage on skin cancer and tanning since 2006; prior studies typically focused on a limited number of publications; and the timeframe analyses were not linked to major public health reports or decisions.

In 2006 the International Agency for Research on Cancer (IARC), the cancer research arm of the World Health Organization (WHO), responded to the alarming increase in skin cancer incidence and the rising popularity of tanning beds by producing a landmark evidence-synthesis report on the dangers of artificial UV exposure and skin cancer [6]. The working group which authored the 2006 IARC report concluded that there was convincing evidence to link indoor tanning bed use and skin cancer (melanoma and squamous cell), a conclusion supported by subsequent systematic reviews [16]. There has been no investigation of the impact of this key report on media coverage of the issues of skin cancer and tanning.

In this study, we analyzed the frequency of coverage of skin cancer and recreational tanning (indoor and outdoor) in popular North American magazines over time (2001 to 2012). In addition to examining volume of coverage, we investigated temporal changes in coverage of skin cancer risk factors, UV behaviors, and early detection information relative to the 2006 IARC report. Based on the significant impact public health reports have had on media coverage of other types of cancer [17], we hypothesized that the effect of the IARC report would be a large increase in the volume of coverage of skin cancer and tanning in popular magazines and corresponding increases in article and image content conveying information about risk factors for skin cancer, encouraging UV protection and UV avoidance, and supporting the importance of early detection.

## Methods

We conducted a directed content analysis on skin cancer and tanning coverage (article text and accompanying images) in 29 high-circulating popular North American magazines between 2001 and 2012. We chose this timeframe because it provided an equal number of years for comparison before and after the 2006 IARC report and was of sufficient duration to allow reliable identification of media reporting patterns. The inclusion of visual data was important because images influence people’s knowledge, attitudes, and behaviors related to their health [18], including skin cancer and tanning [19,20].

Magazines were selected based on circulation numbers, target readership, genre of focus, and whether they were published consecutively during the timeframe of interest. These details were obtained from the *Audit Bureau of Circulations*, *Canadian Advertising Rates and Data*, and magazine media kits. The magazines included in the analysis were women’s (n = 15), men’s (n = 10), female youth (n = 1), and news (n = 3); magazine names and circulation sizes are shown in Table 1. Male youth magazines were not available for the timeframe of interest. Only English language magazines were included.

**Table 1:** Magazine genre, circulation, and number of skin cancer and tanning articles published (2001–2012)

**Country**	**Magazine**	**Type/focus of magazine**	**Circulation (2012)**	**Number (Mean/year, SD)**
United States	*Good Housekeeping*	Women’s general interest	4,346,747	46 (3.8, 2.7)
	*Family Circle*	Women’s general interest	4,100,977	12 (1.0, 0.6)
	*Ladies Home Journal*	Women’s general interest	3,205,302	22 (1.8. 1.8)
	*Cosmopolitan*	Women’s beauty/fashion	3,017,834	65 (5.4, 2.7)
	*Glamour*	Women’s beauty/fashion	2,374,291	25 (2.1, 2.1)
	*Vogue*	Women’s beauty/fashion	1,222,373	16 (1.3, 1.2)
	*Elle U.S.*	Women’s beauty/fashion	1,121,529	48 (4.0, 2.3)
	*Shape*	Women’s health/fitness	1,635,933	112 (9.3, 4.6)
	*Self*	Women’s health/fitness	1,528,583	48 (4.0, 3.7)
	*GQ*	Men’s general interest	963,507	3 (0.3, 0.5)
	*Esquire*	Men’s general interest	721,399	10 (0.8, 1.1)
	*Details*	Men’s general interest	461,937	2 (0.2, 0.4)
	*Sports Illustrated*	Men’s sport/science	3,204,945	1 (0.1, 0.3)
	*Golf Digest*	Men’s sport/science	1,678,538	13 (1.1, 0.7)
	*Popular Science*	Men’s sport/science	1,350,685	7 (0.6, 1.2)
	*Field and Stream*	Men’s sport/science	1,252,833	1 (0.1, 0.3)
	*Men’s Health*	Men’s health/fitness	1,918,387	72 (6.0, 2.6)
	*Men’s Fitness*	Men’s health/fitness	585,265	24 (2.0, 2.0)
	*Muscle and Fitness*	Men’s health/fitness	325,000	17 (1.4, 1.1)
	*Seventeen*	Teen girls’ general interest	2,025,299	33 (2.8. 1.5)
	*Time*	News	3,276,822	16 (1.3, 1.2)
	*Newsweek*	News	1,527,156	15 (1.3, 1.7)
Canada	*Chatelaine*	Women’s general interest	550,613	23 (1.9, 1.3)
	*Canadian Living*	Women’s general interest	511,817	20 (1.7, 1.4)
	*Homemakers*	Women’s general interest	300,764	11 (0.9, 1.1)
	*FASHION*	Women’s beauty/fashion	141,760	32 (2.7, 2.0)
	*Elle Canada*	Women’s beauty/fashion	131,365	18 (1.5, 1.1)
	*Flare*	Women’s beauty/fashion	127,341	43 (3.6, 2.5)
	*Maclean’s*	News	321,095	6 (0.5, 0.9)

Magazines are listed by type/focus (e.g., news magazine) and showing highest to lowest circulation numbers for that type/focus for the year 2012.

Magazines were searched electronically for skin cancer and tanning content through the *Canadian Periodical Index*, *Reader’s Guide to Periodical Literature*, *LexisNexis*, *Factivia*, and *ProQuest Research Library* – and manually using the table of contents for those not indexed online – from January 2001 to December 2012 inclusive. The search terms were: skin cancer, melanoma, basal cell carcinoma, squamous cell carcinoma, (tan* or indoor tan* or outdoor tan* or suntan*), sunburn, sunscreen, and sunblock, together with Boolean operators. Advertisements, articles outside of the date range, or articles which did not have skin cancer and tanning as the main focus were excluded. Magazine article text was retrieved electronically; images were obtained through library archives. Our data (articles and images) came from publicly available archived magazines and, as such, the study did not require university research ethics approval.

We developed a codebook based on skin cancer risk factors, prevention guidelines, and screening information set forth by the American Association of Dermatology, Canadian Dermatology Association, American Cancer Society, Canadian Cancer Society, the WHO, and the IARC. We noted key messages from the 2006 IARC report: UV exposure is linked to skin cancer, people with light skin/hair/eyes are especially at risk, indoor tanning should be avoided, there is a 75% increased risk of melanoma associated with tanning bed use, and sunburn is a risk factor for skin cancer [6]. We also included information about the presence of tanned beauty ideals and tanning behaviors. The presence of tanned beauty ideals was determined based on information in text or images that conveyed a tanned appearance to be attractive or desirable. For text, this meant examining the article for language which promoted a tan (e.g., a tan will make you look better, a tan with give you that healthy glow). For images, we considered pictures of people depicted in favorable or desirable ways (i.e., attractive, to be held in high-regard) with visual evidence of tanned skin to be promoting the tanned beauty ideal. Evidence of a tan was judged on a variety of factors including, for example, visible tan lines and a mismatch between other phenotypic characteristics (light eye and hair color) and skin tone (very bronzed-looking skin). Resulting variables, and a brief description of each, are provided in Table 2. We coded for article date, length, number of images accompanying each article, and the main focus (skin cancer, indoor tanning, outdoor tanning, both indoor and outdoor tanning, self-tanning with lotions/sprays/bronzers, sunscreen, post-sun care, or other). Main focus was determined by the title, by-line, introductory paragraph, and amount of space (at least 75%) within an article devoted to the subject.

**Table 2:** Risk factors, UV behaviors, and early detection variables

**Variable**	**Text description**	**Image description**
**Risk factors**		
UV exposure	Mention of UV exposure (solar or artificial) as risk factor for skin cancer	Depiction of someone who has had skin cancer being exposed to UV radiation
Light skin/hair/eyes	Mention of having susceptible phenotype (light colored hair, skin or eyes) as risk factor for skin cancer	Depiction of person who has had skin cancer with susceptible phenotype
Moles	Mention of increased risk of skin cancer with abnormal moles or more than 50 moles	Depiction of numerous or abnormal moles
History of skin cancer	Mention of having personal or family history of skin cancer as a risk factor	Depiction of recurrence of skin cancer or of family members affected by skin cancer
Sunburns	Mention of sunburn as risk factor for skin cancer	Depiction of someone with sunburn, with some connection to skin cancer
**UV behaviors**		
Tanned look	Promotes tanned ideal or having a tanned look (a tan is beautiful, sexy, or healthy-looking)	Depiction of a person with tanned skin (i.e., image of person depicted in a favorable way who appears to have skin darkened by UV exposure)
Self-tanners	Promotes use of self-tanners (lotions or sprays applied topically to produce appearance of suntan)	Depiction of self-tanner, of someone applying self-tanner, or having a self-tan
Solar UV avoidance	Promotes sun avoidance	Depiction of a person either not exposed to the sun or without suntan
Discourages indoor tanning	Information discouraging the behavior	Negative depiction of indoor tanning (e.g., picture of a tanning bed with an “x” over it)
Encourages indoor tanning	Information encouraging the behavior (e.g., indoor tanning is good for you/provides vitamin D/prevents sunburns)	Positive depiction of someone indoor tanning (e.g., attractive, healthy-looking person in a tanning bed)
Promotes shade	Promotes seeking shade to avoid UV exposure	Depiction of someone seeking shade
Promotes hats	Promotes wearing a hat to protect the face from UV exposure	Depiction of a hat or of someone wearing a hat
Promotes clothes	Promotes use of protective clothing	Depiction of protective clothing or of someone wearing protective clothing
Promotes sunscreen	Promotes use of sunscreen	Depiction of sunscreen or of someone applying sunscreen
SPF level (30+)	Promotes or mentions SPF level of 30 or higher	Depiction of sunscreen visibly labelled with SPF 30 or higher
**Early detection**		
ABCD criteria	Mention and/or description of the ABCD criteria	Depiction of moles which exhibit the ABCD criteria
Skin self-examination	Promotes skin self-checking for skin cancer	Depiction of someone conducting a skin self-examination or example images of dangerous mole characteristics to look for
Physician skin examination	Promotes seeking a physician to do a skin examination for skin cancer	Depiction of someone having a physician-led skin examination conducted

ABCD = asymmetry, border irregularity, color, diameter.

One researcher coded all text and images. To ensure coding reliability, a randomly selected subset of articles (~10%; n = 86) and images (~10%; n = 127) were independently coded by a second researcher and inter-coder reliability calculated; this is a standard methodology and acceptable sample size for establishing inter-coder reliability in media content analyses [21]. Cohen’s kappa scores ranged from 0.83 to 1.00. There were minor discrepancies in how frequently the two researchers coded some of the risk factors for skin cancer. After discussion, it was determined that the discrepancy existed because of differences in interpreting what it meant for an article to state risk. The resolution agreed upon was that risk factors had to be explicitly stated and linked to the disease (e.g., sun exposure increases the chances of getting skin cancer); vague indications of risk without mention of the disease were excluded (e.g., sun exposure is dangerous). The codebook was updated to reflect this decision, which was then used to inform the full dataset.

Data were analyzed (SPSS v22, SPSS Inc., Chicago, IL) using descriptive statistics, including two-way chi-square tests and Pearson correlation coefficients. All statistical tests were two-tailed. We considered statistical results with *p* < .05 to be statistically significant.

## Results

There were 761 articles on skin cancer and recreational tanning published in 29 U.S. and Canadian magazines between 2001 and 2012. Data retrieval was high, with 98% of article text (*n* = 755) and 95% of article images (*n* = 1267) obtained. Articles ranged from 0.25 to 10 pages (*M* = 1.2, *SD* = 1.4) and contained 16 to 4706 words (*M* = 516.1, *SD* = 634.5). Each article had between 0 and 18 accompanying images (*M* = 1.7, *SD* = 1.8). (Note: Analyses on the overall volume of coverage are based on 761 articles. Because six of these articles could not be obtained, analyses involving article content, e.g., risk factor variables, are based on the 755 articles which were retrieved.)

Approximately 80% of articles (*n* = 608) came from U.S. magazines and the remaining 20% (*n* = 153) from Canadian magazines. Articles were from women’s (71%), men’s (20%), female youth (4%), and news (5%) magazines. Table 1 shows the 12 year total and mean number of articles on skin cancer and tanning published each year for each magazine included in the study. The main focus of the articles was: sunscreen use (38%), skin cancer (22%), self-tanning (18%), indoor tanning (6%), outdoor tanning (5%), post-sun care (2%), both indoor and outdoor tanning (2%), or other (<1%).

**Figure 1:**
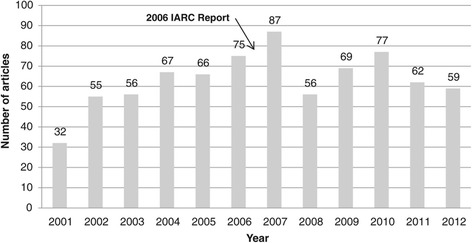
**Skin cancer and tanning articles published per year (2001 to 2012) in 29 magazines.** Note: the numbers of articles published on skin cancer and tanning in 29 popular magazines during each year of the study (2001 to 2012) are represented by the bars.

There were significantly more articles published after (*n* = 410; 54%) compared to before (*n* = 351; 46%) the IARC report (*χ*^2^ = 4.57, *df* = 1, *p* < .05). Figure 1 depicts the frequency of articles about skin cancer and recreational tanning published in the magazines for each year of the study (*M* = 63.4, *SD* = 13.9). The largest volume of articles (*n* = 87) occurred in 2007, the year immediately after the IARC report. Compared to the year immediately preceding the IARC report, this represents an increase of 16% from 2006 to 2007, which was followed by a subsequent drop-off of 36% from 2007 to 2008. As shown in Figure 1, there is a linear increase in the volume of articles on skin cancer and tanning leading up to the IARC report (2001 to 2007), which did not occur in the years after the report (2007 to 2012).

Of the articles published after the IARC report, 3% (*n* = 12) mentioned the IARC report specifically and 7% (*n* = 28) mentioned a key IARC statistic (i.e., risk of melanoma is increased by 75% when the use of tanning devices starts before age 30).

Table 3 outlines the frequency of article text and images conveying each risk factor for skin cancer before vs. after the IARC report. There were no statistically significant differences in the frequency of articles mentioning any of the risk factors for skin cancer before relative to after the IARC report. With respect to images, only susceptible phenotype (light skin/hair/eyes) appeared in a greater percentage of images after compared to before the IARC report.

**Table 3:** Risk factors, UV behaviors, and early detection in magazines before vs after 2006 IARC report

**Variable**	**Content type**	**Before IARC report**		**After IARC report**		***χ*** ^**2**^ **(** ***df*** **= 1) and significance**
	Text (n = 755)	%	(No./349)^a^	%	(No./406)^a^	
	Image (n = 1267)	%	(No./553)^a^	%	(No./714)^a^	
**Skin cancer risk factors**						
UV exposure	Text	39	(137)	40	(163)	0.06, *p* = .803
	Image	4	(21)	3	(21)	0.71, *p* = .398
Light skin	Text	15	(51)	12	(50)	0.86. *p* = .355
	Image	11	(62)	15	(109)	4.39, *p* = .036*
Moles	Text	10	(33)	13	(52)	2.11, *p* = .146
	Image	6	(31)	5	(35)	0.31, *p* = .576
History of skin cancer	Text	8	(28)	10	(41)	0.97, *p* = .324
	Image	0	(0)	0	(0)	n/a
Sunburns	Text	10	(34)	11	(44)	0.24, *p* = .622
	Image	<1	(3)	1	(9)	1.71, *p* = .191
**UV behaviors**						
Tanned look	Text	36	(125)	25	(103)	9.72, *p* = .002**
	Image	45	(250)	37	(262)	9.38, *p* = .002**
Self-tanners	Text	31	(107)	22	(89)	7.46, *p* = .006**
	Image	21	(116)	13	(89)	16.65, *p* < .001***
Solar UV avoidance	Text	19	(65)	12	(48)	6.82, *p* = .009**
	Image	12	(64)	14	(104)	1.82, *p* = .177
Discourages indoor tanning	Text	16	(54)	18	(71)	0.55, *p* = .458
	Image	1	(5)	1	(7)	0.02, *p* = .889
Promotes indoor tanning	Text	2	(6)	2	(7)	0.00, *p* = .996
	Image	2	(10)	3	(14)	0.04, *p* = .843
Promotes shade	Text	8	(28)	8	(33)	0.00, *p* = .958
	Image	8	(46)	7	(48)	1.16, *p* = .282
Promotes hats	Text	16	(54)	14	(58)	0.21, *p* = .647
	Image	10	(53)	9	(61)	0.41, *p* = .521
Promotes clothes	Text	14	(48)	13	(52)	0.15, *p* = .702
	Image	9	(48)	8	(54)	0.53, *p* = .469
Promotes sunscreen	Text	57	(199)	69	(280)	11.55, *p* < .001***
	Image	19	(104)	22	(154)	1.47, *p* = .226
SPF level (30+)^b^	Text	39	(69)	60	(127)	15.65, *p* < .001***
	Image	61	(45)	82	(90)	9.99, *p* = .002**
**Early detection**						
ABCD criteria	Text	5	(19)	7	(30)	1.17, *p* = .279
	Image	2	(10)	2	(11)	0.13, *p* = .724
Skin self-examination	Text	16	(54)	19	(75)	1.19, *p* = .287
	Image	6	(35)	6	(45)	0.02, *p* = .903
Physician-led skin examination	Text	18	(62)	21	(85)	1.20, *p* = .311
	Image	1	(6)	1	(6)	0.20, *p* = .656

* = < .05; ** = *p* < .01; *** = *p* < .001; n/a = not applicable.

^a^Denominator = total number of articles or images on skin cancer and tanning published in the six years before (2001–2006) or after (2007–2012) the IARC report in 29 popular magazines.

^b^Articles and images not indicating a specific SPF level were excluded.

ABCD = asymmetry, border irregularity, color, diameter.

The findings were mixed for changes in the frequency of articles which mentioned, and images which depicted, UV behaviors before compared to after the IARC report (Table 3). After the IARC report there was an increase in the percentage of articles encouraging the use of sunscreen as well as articles and images encouraging the use of high SPF sunscreens (SPF 30 or more). There were decreases in the percentage of articles encouraging solar UV avoidance, articles and images promoting self-tanners, and articles and images promoting the tanned look as desirable after compared to before the IARC report.

There were no significant differences in the percentage of content focusing on any of the early detection or screening variables (i.e., mention/depiction of ABCD criteria, encouraging skin self-exams, or encouraging physician-led skin exams) before compared to after the IARC report (Table 3).

We conducted correlation analyses between the years and the volume (number) of articles reporting on each of the variables of interest in order to look for patterns in coverage over time. No significant relationships between year and any of the risk factor or early detection variables in article text were found; in contrast, significant patterns of coverage over time were detected for three UV behavior variables. As the years progressed, the number of articles discouraging indoor tanning bed use increased (*r*(12) = 0.603, *p* < .05), articles encouraging sunscreen use (*r*(12) = 0.710, *p* < .01) and promoting high sun protection factor (SPF) sunscreen (*r*(12) = 0.860, *p* < .01) also increased.

**Figure 2:**
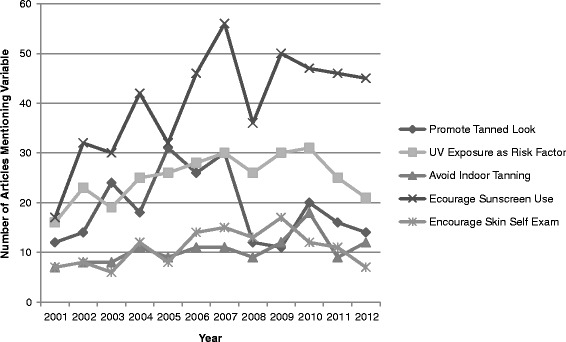
**Representative study variables mentioned in article text (2001–2012).** Note: Five representative study variables were selected to be displayed: one risk factor variable (UV exposure); three UV behavior-related variables (promotes tanned look, discourages indoor tanning, encourages sunscreen use); and one early detection variable (encourages skin self-examination). These variables were selected based on: their significance to skin cancer risk, prevention, and early detection; their specific mention in the IARC report; and, in the case of “tanned look”, because a tanned appearance is a primary reason for indoor tanning bed use.

**Figure 3:**
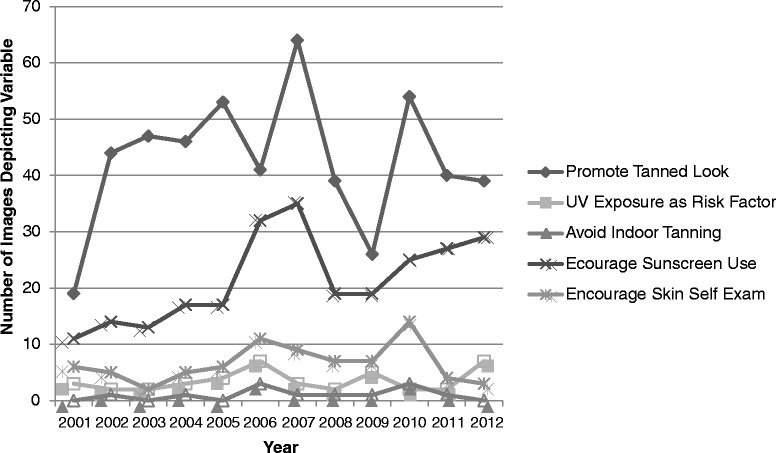
**Representative study variables depicted in article images (2001–2012).** Note: Five representative study variables were selected to be displayed: one risk factor variable (UV exposure); three UV behavior-related variables (promotes tanned look, discourages indoor tanning, encourages sunscreen use); and one early detection variable (encourages skin self-examination). These variables were selected based on: their significance to skin cancer risk, prevention, and early detection; their specific mention in the IARC report; and, in the case of “tanned look”, because a tanned appearance is a primary reason for indoor tanning bed use.

Similar to text, there were also no significant correlations between year and number of images on risk factor or early detection variables. For UV behavior variables, however, images more frequently promoted sunscreen use (*r*(12) = 0.669, *p* < .05) and higher SPF sunscreens (*r*(12) = 0.892, *p* < .01) over the course of the study timeframe. Figures 2 and 3 illustrate the relationships between the number of articles reporting, and images depicting, representative skin cancer and tanning variables (UV exposure as a risk factor for skin cancer, encouraging sunscreen use, discouraging indoor tanning, promoting the tanned look, and encouraging skin self-examination) over the 12 years of data included the study.

## Discussion

This is the largest and most comprehensive study to date on popular North American magazine coverage of skin cancer and tanning. It is also the first to examine temporal comparisons relative to a key global public health document on skin cancer. Consistent with our hypothesis, the volume of skin cancer and recreational tanning coverage in popular magazines increased, albeit modestly, after the 2006 IARC report. In the year following this report, there was a small spike in coverage (16%) followed by a subsequent drop-off. This suggests that the report had a temporary and only modest impact on the amount of coverage immediately following its release. This is consistent with the idea that heightened attention to an issue in the media is infrequently sustained for a significant amount of time – a phenomenon known as the “issue attention cycle” [22]. The fairly steady increase in the number of articles on skin cancer and tanning between 2001 and 2007 may have reflected a growing public health interest in, and knowledge about, the issue of skin cancer and the dangers of UV exposure. The 2006 IARC report is an evidence-synthesis report and thus, it is possible that leading up to it, there was accumulating research evidence and heightened concern by public health professionals, which was picked up by the media. The lack of a linear increase in the volume of articles from 2007 to 2012 (post-IARC report) may be related to other public health reports or decisions relevant to skin cancer, or other factors not examined in the present study.

Contrary to our second hypothesis, we found little focus on the important messages from the 2006 IARC report in articles and accompanying images published in 29 popular North American magazines. Few articles mentioned the report by name or stated the key report statistic (i.e., 3% mentioned the report, 7% mentioned the key IARC statistic) which was highlighted in the report press release. The only skin cancer risk factor which appeared to be affected by the report was susceptible phenotype, which was present in images to a greater extent after the report. The evidence synthesized in the report pertained to light-skinned populations, so this is potentially a positive sign. Nevertheless, article text was not influenced in the same way about susceptible phenotype, suggesting uptake of this message was limited and the message was mixed (no substantial increase in reporting in text but a greater depiction in images). Further, there was no change in the percentage of articles mentioning UV exposure as a risk factor and no change in the percentage of articles or images discouraging indoor tanning, even though these were primary messages of the report.

There were inconsistent changes in magazine coverage for UV behaviors over time, despite our hypothesis of an increase for all behavioral variables. On the positive side, there was a greater percentage of content encouraging sunscreen use and promoting higher SPF sunscreens after the 2006 IARC report. Indeed, sunscreen use was the only skin cancer prevention strategy reported more frequently over the study timeframe. While generally positive that magazines encourage sunscreen use, as it is a recommended method for prevention of skin cancer [4], IARC identifies sunscreen as a *secondary* form of protection against skin cancer, *after* sun avoidance, shade-seeking, and protective clothing [23]. We speculate that the report was not the impetus for the increase in images and articles encouraging sunscreen, but rather may reflect a growing commercial market for sunscreen products. It would be interesting to know if the message about sunscreen use in article content was associated with a simultaneous increase in sunscreen advertisements, as advertising and editorial content in magazines are often related [24]. This will be an important area for future research. While the promotion of sunscreen use increased, the percentage of articles discouraging artificial UV exposure did not change and the percentage discouraging solar UV exposure decreased. Taken together, our results suggest that the magazines included in this study shifted from the “message” of UV avoidance to one of promotion of UV protection.

It is encouraging that the percentage of articles and images promoting the tanned look as desirable in popular magazines decreased after the 2006 IARC report. People are especially influenced by images accompanying media articles [25] and images of attractive people with suntans increase the social desirability of a suntan and encourage UV exposure [26], so this is a positive change. The data also show that the number of articles and images encouraging the use of self-tanners decreased after the report. The reason for this decrease in coverage for self-tanners is not evident from our data but may reflect the lesser emphasis on the tanned look. At the same time, however, it suggests readers were less frequently messaged about alternatives to achieving a tan through recreational UV exposure. Although the promotion of the tanned look as ideal decreased after 2006, it still was common throughout the study timeframe. This may partly explain why researchers have found magazine use to be associated with reduced behavioral control to avoid unprotected UV exposure [27].

There were no changes in the percentage of articles or images with content about early detection after the 2006 IARC report. Given the report does not specifically focus on early detection, this is not surprising. Nevertheless, the report emphasized the increased risk of skin cancer from indoor tanning and those exposed should be especially mindful of screening. If people are not receiving information about the importance of early detection from the popular media, it is unclear where or how they might receive this information.

The small impact of the IARC report on the proportion of text and image messages in leading popular magazines about recreational UV exposure as a risk factor for skin cancer, on discouraging indoor tanning, and on other skin cancer risk factors and behaviors, is puzzling and alarming. The limited influence of this seminal skin cancer and recreational tanning report on frequency of popular magazine coverage of related health issues is in stark contrast to the enormous effect of the analogous U.S. Surgeon General’s [28] report linking tobacco smoking and lung cancer. In a study examining coverage of smoking and health in magazines between 1950 and 1983, the largest spike in magazine coverage occurred in the year immediately following the 1964 Surgeon General’s report, when the volume of coverage increased by approximately 107% [17]. The increased media coverage was associated with smoking cessation [29].

It is challenging to interpret why there was only a very modest impact of the IARC report on skin cancer coverage in popular mass magazines, given that recreational tanning leads to more cases of skin cancer than smoking does to lung cancer [8]. The lack of skin cancer coverage relative to lung cancer coverage after milestone reports could relate to a number of factors including: social stigma around smoking [29] which is not present for tanning; the status of the tan as a Western beauty ideal [30]; tobacco companies being large multi-national corporations and thus easier for the media to target as the “bad guy” compared to small-business tanning salons [31]; or possible differences in the reach or influence of Surgeon General’s reports compared to IARC reports in setting the North American news agenda. Thus, it will be important to follow up the current study with an evaluation of the frequency and types of skin cancer and tanning messages in popular magazines relative to the newly released 2014 U.S. Surgeon General’s report on skin cancer prevention [32].

Another possible explanation for the lack of change in media coverage could relate to the press release for the 2006 IARC report [33] which stated “sunbed use in youth unequivocally associated with skin cancer” but also that “studies do not provide consistent evidence that use of indoor tanning facilities in general is associated with […] skin cancer”. Although there were explanations for these conclusions, they may have been interpreted as contradictory by journalists and confusing for the public who receive skin cancer risk messages through popular media outlets. This highlights the challenge of balancing scientifically accurate information and readily comprehensible skin cancer information packaged for public dissemination.

This study is not without limitations. Our data consisted of high-circulating magazines with considerable reach; however, we did not include other types of print media, such as newspapers, which may have been influenced differently by the IARC report. Additional types of information about skin cancer and UV exposure, such as promoting UV exposure for Vitamin D production, could also be collected in future work. We also did not investigate magazine advertisements, but this is worthy of study in further research. While every effort was made to be objective in the analysis by clearly operationalizing each variable in the codebook and performing inter-rater reliability checks, there is an inherent subjectivity with analyzing visual content. This was especially true regarding coding for whether a person was depicted with tanned skin or not. Other studies have also coded images for similar variables and share the same limitations [34]. We used the 2006 IARC report as the focus of the comparison and noted relationships; however, we do not know that this report had a causal effect on the changes in the article text and images noted in the findings. Finally, we did not assess whether readers’ knowledge and behavior are influenced by this magazine content.

## Conclusions

The 2006 IARC report appears to have had a small and limited impact on the frequency of coverage on skin cancer and recreational tanning as topics in popular North American magazines; key messages from the IARC report were not picked up by popular magazines (e.g., UV exposure as a risk factor, avoid indoor tanning). There were no changes in terms of magazine reporting on skin cancer risk factors or early detection strategies. A few behavioral variables changed over the 12 years of study, including an increased emphasis on sunscreen use and a decreased emphasis on the attractiveness of a tanned appearance. Magazine coverage of most of the other behavioral variables was flat over time. Public health educators will need to consider ways to improve or supplement the dissemination of skin cancer information from public health reports into the mass media, including developing partnerships with magazine journalists and editors in social marketing of this important public health issue.

## Abbreviations

UV: Ultraviolet
IARC: International Agency for Research on Cancer
WHO: World Health Organization
US: United States
SPF: Sun protection factor
ABCD: Asymmetry, border, color, diameter

## References

[1] World Health Organization: Skin cancers. http://www.who.int/uv/faq/skincancer/en/index1.html.

[2] American Cancer Society: What are the key statistics about melanoma skin cancer? http://www.cancer.org/cancer/skincancer-melanoma/detailedguide/melanoma-skin-cancer-key-statistics.

[3] Canadian Cancer Society: Canadian Cancer Statistics. http://www.cancer.ca/en/cancer-information/cancer-101/canadian-cancer-statistics-publication/?region=on.

[4] American Academy of Dermatology: How do I prevent skin cancer? http://www.aad.org/spot-skin-cancer/understanding-skin-cancer/how-do-i-prevent-skin-cancer.

[5] Ries LAG, Melbert D, Krapcho M, Mariotto A, Miller BA, Feuer EJ, et al. SEER Cancer Statistics Review. 1975–2004. Bethesda: National Cancer Institute. http://seer.cancer.gov/csr/1975_2004/.

[6] International Agency for Research on Cancer: IARC Working Group Reports Volume 1: Exposure to Artificial UV Radiation and Skin Cancer. Lyon: World Health Organization and IARC. 2006. http://www.iarc.fr/en/publications/pdfs-online/wrk/wrk1/ArtificialUVRad&SkinCancer.pdf.

[7] Heckman CJ, Coups EJ, Manne SL (2008). Prevalence and correlates of indoor tanning among US adults. J Am Acad Dermatol.

[8] Wehner MR, Chren MM, Nameth D, Choudhry A, Gaskins M, Nead KT (2014). International prevalence of indoor tanning: a systematic review and meta-analysis. JAMA Dermatol.

[9] Eadie D, MacAskill S (2007). Results from an exploratory study of sun protection practice: implications for the design of health promotion messages. Health Educ.

[10] Dixon H, Warne C, Scully M, Dobbinson S, Wakefield M (2014). Agenda-setting effects of sun-related news coverage on public attitudes and beliefs about tanning and skin cancer. Health Commun.

[11] Sinclair CA, Makin JK, Tang A, Brozek I, Rock V (2014). The role of public health advocacy in achieving an outright ban on commercial tanning beds in Australia. Am J Public Health.

[12] Cho H, Hall JG, Kosmoski C, Fox RL, Mastin T (2010). Tanning, skin cancer risk, and prevention: a content analysis of eight popular magazines that target female readers: 1997–2006. Health Commun.

[13] Stryker JE, Solky BA, Emmons KM (2005). A content analysis of news coverage of skin cancer prevention and detection, 1979 to 2003. JAMA Dermatol.

[14] Heneghan MK, Hazan C, Halpern AC, Oliveria SA (2007). Skin cancer coverage in a national newspaper: a teachable moment. J Cancer Educ.

[15] Scully M, Makin J, Maloney S, Wakefield M (2014). Changes in coverage of sun protection in the news: threats and opportunities for emerging issues. Health Educ Res.

[16] Boniol M, Autier P, Boyle P, Gandini S (2012). Cutaneous melanoma attributable to sunbed use: review and meta-analysis. BMJ.

[17] Pierce JP, Gilpin EA (2001). News media coverage of smoking and health is associated with changes in population rates of smoking cessation but not initiation. Tob Control.

[18] Houts PS, Doak CC, Doak LG, Loscalzo MJ (2006). The role of pictures in improving health communication: A review of research on attention, comprehension, recall, and adherence. Patient Educ Couns.

[19] McWhirter JE, Hoffman-Goetz L (2013). Systematic review of population based studies on the impact of images on UV attitudes and behaviours. Health Promot Int.

[20] McWhirter JE, Hoffman-Goetz L (2013). Visual images for patient skin self-examination and melanoma detection: A systematic review of published studies. J Am Acad Dermatol.

[21] Riffe D, Lacy S, Fico F (2013). Analyzing Media Messages: Using Quantitative Content Analysis in Research.

[22] Downs A (1972). Up and down with ecology: the issue attention cycle. Public Interest.

[23] Vainio H, Bianchini F (2001). Handbook of cancer prevention.

[24] Warner KE, Goldenhar LM, McLaughlin CG (1992). Cigarette advertising and magazine coverage of the hazards of smoking. A statistical analysis. N Engl J Med.

[25] Messaris P, Abraham L, Reese S, Gandy O, Grant A (2001). The role of images in framing news stories. Framing Public Life.

[26] Dixon HG, Warne CD, Scully ML, Wakefield MA, Dobbinson SJ (2011). Does the portrayal of tanning in Australian women’s magazines relate to real women's tanning beliefs and behavior?. Health Educ Behav.

[27] Lovejoy J, Riffe D, Lovejoy TI (2015). An examination of indirect effects of exposure and attention to health media on intentions to avoid unprotected sun exposure. Health Commun.

[28] U.S. Department of Health, Education, and Welfare: Smoking and Health: Report of the Advisory Committee of the Surgeon General of the Public Health Service. Washington: Public Health Service, U.S. Government Print Office. 1964. http://www.surgeongeneral.gov/library/reports/index.html.

[29] Alamar B, Glantz S (2006). Effect of increase social unacceptability of cigarette smoking on reduction in cigarette consumption. Am J Public Health.

[30] Hunt Y, Augustson E, Rutten L, Moser R, Yaroch A. History And Culture Of Tanning In The United States. In Shedding Light On Indoor Tanning. Edited By Heckman CJ, Manne SL. New York. Springer Science+Business Media; 2012. http://www.springer.com/biomed/cancer/book/978-94-007-2047-3.

[31] Group ITW (2011). Report of the Indoor Tanning Working Group.

[32] U.S. Department of Health and Human Services: The Surgeon General’s Call to Action to Prevent Skin Cancer. Washington: U.S. Department of Health and Human Services, Office of the Surgeon General. 2014. http://www.surgeongeneral.gov/library/calls/prevent-skin-cancer/call-to-action-prevent-skin-cancer.pdf. 25320835

[33] IARC Press Release 171: Sunbed use in youth unequivocally associated with skin cancer. http://www.iarc.fr/en/media-centre/pr/2006/pr171.html.

[34] Chapman S, Marks R, King M (1992). Trends in tans and skin protection in Australian fashion magazines, 1982 through 1991. Am J Public Health.

